# Identification of Tie2 as a sensor for reactive oxygen species and its therapeutic implication

**DOI:** 10.1016/j.redox.2025.103555

**Published:** 2025-02-20

**Authors:** An Vuong Quynh Pham, Yongwoo Na, Gyeongseo Suk, Chansik Yang, So Min Kang, Joonha Lee, Hongseo Choi, Wook Kim, Sung Wook Chi, Sangyeul Han, Hae Woong Choi, Hyeonwoo Kim, Chungho Kim

**Affiliations:** a Department of Life Sciences, Korea University, Seoul, 02841, Republic of Korea; b Department of Biological Sciences, Korea Advanced Institute of Science and Technology, Daejeon, 34141, Republic of Korea; c Department of Molecular Science & Technology, Ajou University, Suwon, 16499, Republic of Korea; d Ingenia Therapeutics, 34 Coolidge Ave. 2nd Floor, Watertown, MA, 02472, United States; e KAIST Stem Cell Center, Korea Advanced Institute of Science and Technology, Daejeon, 34141, Republic of Korea

**Keywords:** Reactive oxygen species, Hydrogen peroxide, Tie2, Inflammation, Psoriasis

## Abstract

Psoriasis is a chronic inflammatory disease characterized by hyperproliferation of keratinocytes and abnormal blood vessels. As hyperproliferation is driven by pro-inflammatory cytokines produced by activated immune cells, therapeutic strategies often target these cytokines to manage the disease. However, the role of abnormally developed blood vessels has often been overlooked in treatment approaches. In this study, we focused on blood vessels in psoriatic lesions and investigated the potential interplay between immune and endothelial cells by adopting imiquimod treated mice as *in vivo* model, together with various cell biological, biochemical, and structural analyses. We found that activated immune cells can generate reactive oxygen species, subsequently inducing oxidative stress in endothelial cells. Oxidative stress impairs endothelial cell layer integrity, thereby facilitating transendothelial migration of immune cells. Mechanistically, oxidative conditions inhibit Tie2 activation, potentially by modifying its cysteine residues, leading to deactivation of its vessel-stabilizing functions. Additionally, we demonstrated that reactivating Tie2 under such conditions could restore endothelial barrier function and alleviate the disease. These results suggest that Tie2 serves as a receptor that is directly responsive to oxidative environments, thereby modulating its kinase activity. Furthermore, we suggest that Tie2 reactivation is a promising alternative therapeutic approach for psoriasis.

## Highlights

• Tie2 phosphorylation can be reduced in vessels within an inflammatory environment.
• Hydrogen peroxide can directly act on cysteine residues in the Tie2 kinase domain, leading to its inactivation.
• Activation of Tie2 can overcome the detrimental effects of oxidative stress both *in vitro* and *in vivo.*

## Graphical abstract

**Figure:**
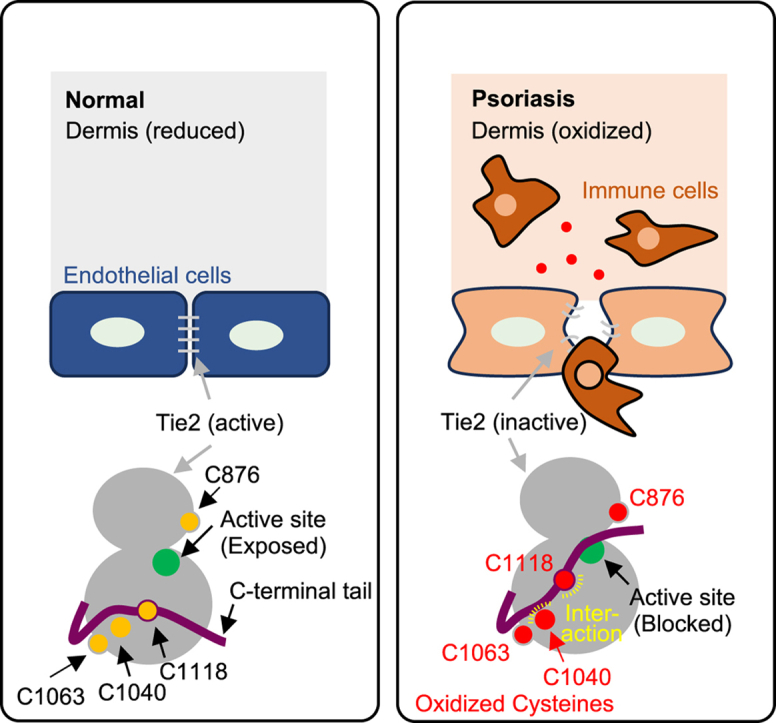

## 1 Introduction

Psoriasis is a skin disease that affects approximately 2 % of the global population [1]. Although not deadly, the disease often has a negative impact on the patients’ work, emotions, and relationships [2]. The most common form of psoriasis is chronic plaque psoriasis (*psoriasis vulgaris*), which is characterized by patches of red, scaly, and thick skin composed of hyperproliferative keratinocytes accompanied by itching, burning, or soreness [3]. The initial stage of this skin disease is triggered by an inflammatory event in which keratinocytes are stimulated to produce pro-inflammatory cytokines, such as tumor necrosis factor (TNF)-α, interleukin (IL)-6, and IL-1 [[4], [5], [6], [7]]. These cytokines activate dermal dendritic cells, thereby elevating the production of IL-12/IL-23 to promote the recruitment of T cells and their subsequent differentiation into T helper 1 (Th1) and Th17 subsets [[8], [9], [10]]. IL-17 and IL-22 produced by these Th cells cause hyperproliferation of keratinocytes [[11], [12], [13]], which in turn secrete IL-8 and antimicrobial peptides to recruit neutrophils [14,15]. Subsequently, the infiltrated neutrophils produce a protease that can cleave IL-36 and release it in its active form [16], amplifying the inflammatory conditions in psoriatic skin lesions [17]. Thus, the abundance of infiltrated neutrophils in lesions indicates the installation of inflammation-amplifying loops and can be considered a hallmark of psoriasis [18,19].

Currently, treatments for mild psoriatic cases focus on inhibiting the hyperproliferation of keratinocytes using retinoids and vitamin D3 analogues [20]. In moderate-to-severe cases, biologics, such as antibodies targeting IL-17 (e.g., secukinumab, ixekinumab, and brodalumab) to hinder keratinocyte hyperproliferation or IL-23 (e.g., ustekinumab, guselkumab, risankizumab, and tildrakizumab) to reduce Th17 cell activation, are considered. Additionally, antibodies against pro-inflammatory cytokine, including TNF-α (e.g., infliximab, adalimumab, certolizumab pegol, and etanercept) or IL-36 (e.g., spesolimab), are currently used to block the amplification of inflammation [21,22]. However, anti-immune therapies often lead to immune-related complications. For example, although anti-IL-17 antibodies effectively treat psoriasis, they may also lead to inflammatory bowel disease or fungal infections [22,23]. Similarly, targeting TNF-α can increase the risk of tuberculosis [24]; anti-IL-23 drugs may cause hidradenitis suppurativa or arthritis; and medications, such as adalimumab (anti-TNF-α), secukinumab (anti-IL-17), and ustekinumab (anti-IL-23), can contribute to the development of thrombocytopenia [[25], [26], [27]]. Considering the complications and side effects associated with current medications used, safer alternative methods are required to treat this disease.

In most histological analyses, thickened patches of keratinocytes in psoriatic lesions appear as long rete ridges, with capillaries situated very close to these ridges [28]. Thus, the proximity of capillaries to the ridges is considered a hallmark of psoriasis [29]. However, the pathophysiological significance of vessels within psoriatic lesions remains largely underexplored. To identify an alternative therapeutic approach for psoriasis, we investigated the potential interplay between these vessels and the considerable influx of immune cells recruited to the psoriatic lesions. Tie2, a representative receptor tyrosine kinase expressed almost exclusively in endothelial cells [30]. When activated by its agonistic ligand, angiopoieitin-1, its tyrosine residues, including Tyr992 in its activation loop, are phosphorylated to induce signaling for vessel stability, while its inhibition results in vessel leakage, for example, during inflammation [31]. Consistently, the artificial activation of Tie2 has been shown to alleviate sepsis progression by preserving endothelial integrity [32]. Therefore, we hypothesized that immune cells recruited in the psoriatic lesion may control Ang1-Tie2 axis in nearby blood vessels to modulate the endothelial integrity for enhancing further recruitment of immune cells. In this study, we suggest that immune cells can induce oxidative conditions, enhancing vessel permeability through the inactivation of Tie2. Given that increased vessel permeability can further recruit immune cells and perpetuate a vicious cycle that worsens the disease, we propose that activating Tie2 stress could represent another approach for future psoriasis treatment strategies.

## 2 Materials and methods

### 2.1 Psoriasis mouse model

Eight-week-old C57BL/6 female mice were purchased from Raonbio Inc. (Yongin, Republic of Korea) and topically applied with 62.5 mg Aldara cream (5 % Imiquimod - IMQ) daily. When needed, mice were injected subcutaneously on their backsides with 100 μL phosphate-buffered saline (PBS) or IGN-002 (10 mg/kg) every two days (days 1, 3, 5, and 7). Images of the mice were taken daily to calculate the Psoriasis Area Severity Index (PASI) score [33]. On day 8, their skin was harvested and cryo-embedded in an O.C.T compound. All experiments on animals complied with the protocols approved by Korea University Institutional Animal Care and Use Committee (approval number 2021–0035, 2023–0096 and 2024–0002).

### 2.2 Cell culture

HEK293T, Chinese hamster ovary [34], and THP-1 cells were purchased from American Type Culture Collection and maintained in Dulbecco's modified Eagle's and RPMI-1640 media, respectively, supplemented with 10 % fetal bovine serum (FBS) and 1 % penicillin-streptomycin at 37 °C using a 5 % CO_2_ incubation chamber. Human umbilical endothelial cells (HUVECs) were purchased from Lonza (Basel, Switzerland) and maintained in Endothelial Basal Medium-2 (EBM) supplemented with EGM-2 SingleQuot (Lonza). For differentiation of THP-1 cells into macrophage-like cells (Mφ), the cells were treated with 400 nM phorbol 12-myristate 13-acetate (PMA) for 48 h.

### 2.3 cDNAs, antibodies, and reagents

Human Tie2 cDNA and FLAG-Tie2ΔECD were prepared, as previously described [35], and the mouse Tie2 cDNA constructed in a similar manner. Cysteine mutations were introduced via site-directed mutagenesis. Anti-mouse CD3 (Ab16669; Abcam, Cambridge, UK), anti-mouse Ly6G (Ab25377; Abcam), anti-4-hydroxy-2-nonenal (4-HNE) (bs-6313R-TR; Bioss, Woburn, MA, USA), anti-mouse and anti-human pTie2 (AF-2720-SP; R&D Systems, Minneapolis, MN, USA), anti-human pTie2 (Ab151704; Abcam), anti-mouse PECAM-1 (550274; BD Biosciences, Franklin Lakes, NJ, USA), anti-human-VE-cadherin (BD Biosciences), anti-PTPRZ (610180, BD Biosciences), rhodamine phalloidin (R415; Thermo Fisher Scientific, Waltham, MA, USA), anti-FLAG (F1804; Sigma-Aldrich, St. Louis, MO, USA), anti-Tie2 (extracellular domain) clone Ab33 (05–584; Merck Millipore, Burlington, MA, USA), and anti-Tie2 (intracellular domain) (Sc-324; Santa Cruz Biotechnology, Dallas, TX, USA) used in this study are commercially available. The Tie2-activating antibody, IGN-002, was provided by Ingenia Therapeutics (Watertown, MA, USA). Ang1 (923-AN-025) was purchased from R&D Systems, and 2′,7′-dichlorodihydrofluorescein diacetate (H_2_DCFDA) (D399), lipopolysaccharide (LPS) (L3012), and *N*-acetyl-l-cysteine [34] (A9165) from Sigma-Aldrich.

### 2.4 Histochemistry

Cryosections of skin samples at a 16-μm thickness were incubated in blocking solution (3 % bovine serum albumin, 5 % normal goat serum, 5 % MOM blocking reagents [MKB-2213-1; Vectorlab, Newark, CA, USA], 0.4 % Triton X-100 in Tris-buffered saline, pH 7.8). The sections were stained and mounted using a fluorescent mounting medium (S302380-2; Dako, Glostrup, Denmark) containing Hoechst. Images were captured using a confocal microscope (LSM800; Zeiss, Oberkochen, Germany) at a 40 × magnification, using the auto-stitching function. Image processing and fluorescence quantification were performed using Zeiss Zen software.

### 2.5 Cell imaging

The confluent HUVEC monolayer was starved of serum for 6 h with 0.5 % FBS and then treated with Ang1 (250 ng/mL), IGN-002 (20 μg/mL), and/or 1 mM H_2_O_2_ for 30 min. The cells were then fixed, stained, and mounted. Images were captured using a fluorescence microscope (Ti-E; Nikon, Tokyo, Japan) under a 100 × magnification lens and processed using NIS-Element AR (Nikon). For co-culture experiments, a confluent monolayer of HUVECs in 6-well plates was incubated in 3 mL EBM with Hoechst for 5 min. THP-1 or Mφ cells were added to the HUVECs at a final concentration of 2.5 × 10^4^ cells/mL, along with H_2_DCFDA (10 μM). For immune cell stimulation, LPS (1 μg/mL) was included. Images of the co-culture were captured every 5 min for a total of 30 minnutes.

### 2.6 Statistical analysis

Data analysis and graph generation were performed using GraphPad Prism (v. 5.03). Statistical significance was determined using the unpaired Student's t-test and one-way or two-way ANOVA with Bonferroni's comparison test as described in the figure legends. Dots on the graphs quantifying histology images represent auto-stitched images, each containing 3 × 3 microscopic fields from the same tissue section. For individual mice, 1–3 auto-stitched images were analyzed, as noted in the figure legends.

## 3 Results

### 3.1 Treatment with imiquimod induces oxidative conditions in mouse skin

**Fig. 1:**
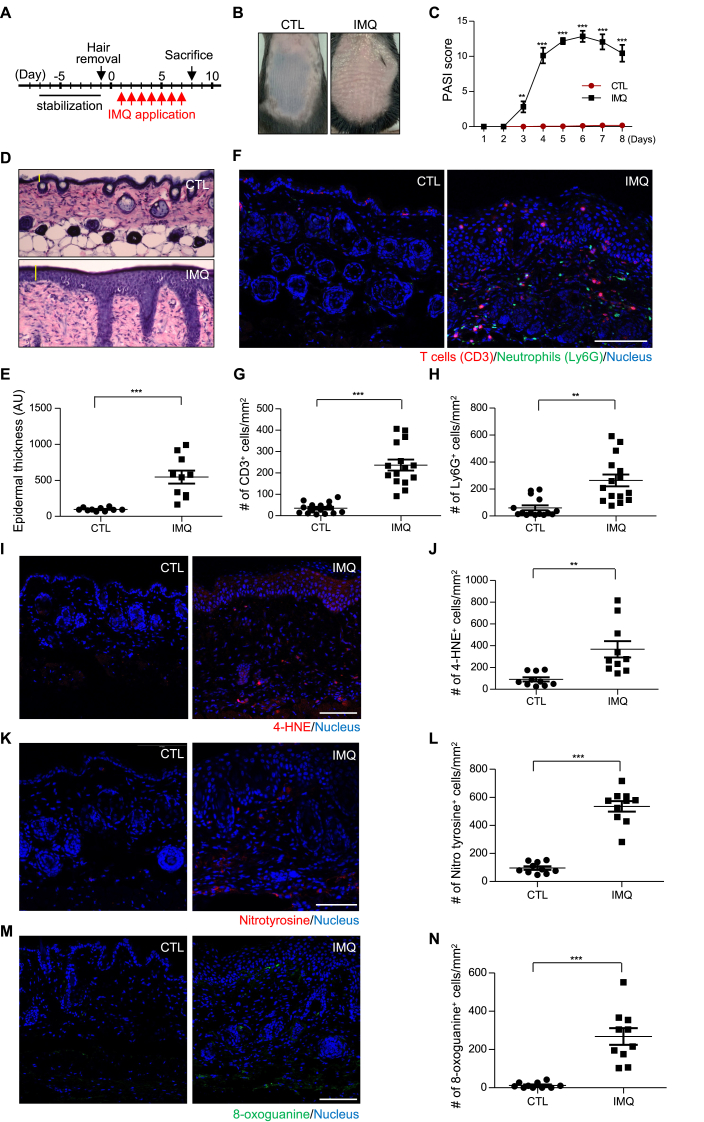
**Psoriasis mouse model shows skin inflammation accompanied by oxidative stress.** (A) Experimental scheme of the seven-day application of imiquimod (IMQ) on the shaven backs of mice to induce a psoriasis-like condition. (B) Back skin of mice treated with either control cream (CTL) or IMQ for seven days. (C) Psoriasis Area and Severity Index (PASI) scores of CTL- and IMQ-treated mice during the treatment period. Error bars indicate the mean ± SEM (n = 5 mice). ∗∗p < 0.01, ∗∗∗p < 0.001 (two-way ANOVA with Bonferroni's comparison test). (D) Hematoxylin and eosin staining of harvested back skin after seven-day treatment, with the epidermis indicated by yellow vertical lines. (E) Epidermal thickness, measured as the area of the epidermis within a microscopic field, is shown in arbitrary units (AU). Each dot represents a microscopic field of a skin section. For each condition, a total of 10 samples from 5 different mice were analyzed, with 2 samples per mouse. (F) Representative images of skin sections of CTL- and IMQ-treated mice stained with anti-CD3 (red), anti-Ly6G (green), and Hoechst. Scale bar, 100 μm. (G, H) The numbers of CD3^+^ and Ly6G^+^ cells were counted and divided by the total area of stained tissue in different auto-stitched images in each condition. For each condition, a total of 15 samples from 5 mice were analyzed, with 3 samples per mouse. (I, K, M) Skin sections stained with 4-hydroxynonenal (4-HNE), nitrotyrosine, and 8-oxoguanine. (J, L, N) The densities of Ly6G^+^ cells, nitrotyrosine^+^ cells, and 8-oxoguanine^+^ cells in (I), (K), and (M), respectively, were measured as described in (G) and (H). For each condition, a total of 10 samples from 5 different mice were analyzed, with 2 samples per mouse. For (E), (G), (H), (J), (L), and (N), data are shown as mean ± SEM. ∗∗p < 0.01, ∗∗∗p < 0.001 (unpaired *t*-test). Scale bar, 100 μm.

To establish a mouse model of psoriasis, we applied treatment with IMQ, a reagent known to induce psoriasis-like conditions in mice via TLR7/8 activation [36], daily for a week on the shaved backs of mice (Fig. 1A). Skin phenotypes were monitored daily before the application of IMQ, with moisture cream-treated mice as a control (Supplementary Fig. S1). On day 8, the control mice had normal skin with hair growth, whereas the IMQ-treated mice appeared to have the characteristic redness and scaly skin patches found in psoriatic lesions (Fig. 1B). The PASI score, which was blindly evaluated, peaked in IMQ-treated mice on days 5 and 6 (Fig. 1C). In contrast, the control mice exhibited no signs of psoriasis. Histological examination of skin sections from day 8 mice stained with hematoxylin and eosin demonstrated notable thickening of the epidermal layer in IMQ-treated mice compared to that in control mice (Fig. 1D–E). When skin sections from control mice were stained using anti-CD3, a pan-marker of the T cell population, and anti-Ly6G antibody, a marker of neutrophils, T cells were exclusively observed in the epidermis, whereas neutrophils were absent (Fig. 1F, left). In contrast, in the psoriasis mouse model, both T cells and neutrophils were notably present, particularly in the dermal area (Fig. 1F, right), indicating a pro-inflammatory state within the psoriatic lesion. The unexpectedly lowered PASI score after the peak at day 5 (Fig. 1C) seems to be caused by change in immune cell profile during disease progression; that is, Ly6G-positive cells, but not CD3-positive cells, were increased at day 5 (Supplementary Fig. S2), with both cell types enriched at day 8 (Fig. 1F–H). The increased number of immune cells in lesions of the psoriasis model (Fig. 1G–H) suggested that these immune cells might induce oxidative conditions within the lesion, as observed in other inflammatory diseases [[37], [38], [39]]. Indeed, immune cells are known to produce various reactive oxygen species (ROS), such as superoxide anion, hydrogen peroxide, hypochlorous acid, or hydroxyl radical for innate immune response [40,41]. 4-HNE, generated by lipid peroxidation under oxidative condition, reacts with biomolecules and, thus, the presence of HNE in cells can be used as an oxidative stress biomarker [42]. Staining of the skin with an antibody recognizing 4-HNE revealed that skin sections from control mice exhibited minimal 4-HNE staining, whereas those from IMQ-treated mice displayed an increased number of 4-HNE-positive cells in both the epidermal and dermal layers (Fig. 1I–J). Similarly, protein oxidation measured by anti-nitrotyrosine antibody and DNA oxidation measured by anti-8-oxoguanine antibody were also increased in the lesion (Fig. 1K–N). This suggests that the psoriatic lesions were in an oxidative environment, possibly resulting from an increased number of immune cells.

### 3.2 Pro-inflammatory immune cells can increase intracellular ROS levels in endothelial cells

**Fig. 2:**
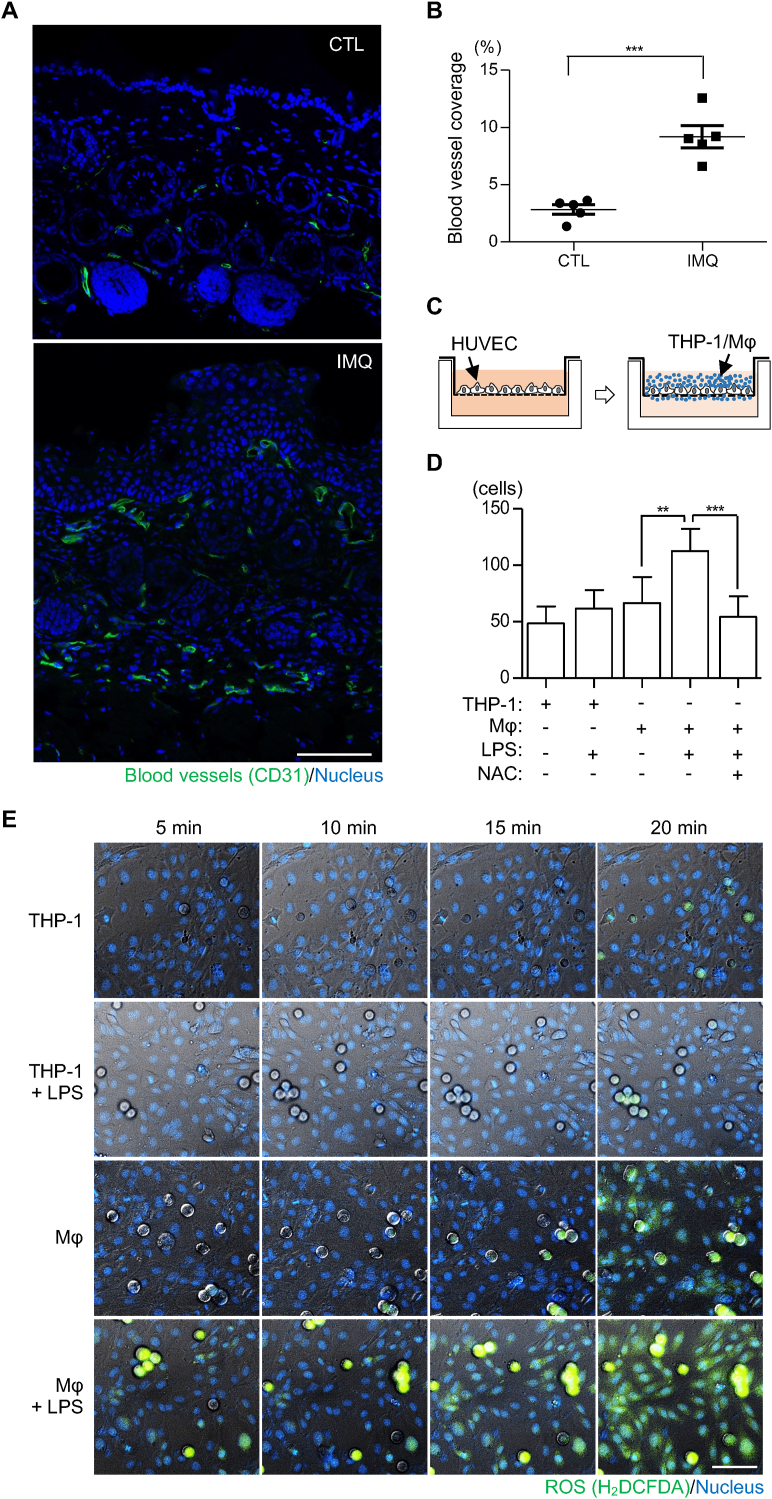
**Pro-inflammatory immune cells can disrupt endothelial barrier function.** (A) Representative images of skin sections from CTL- and IMQ-treated mice stained with anti-CD31 (green) and Hoechst. Scale bar, 100 μm. (B) Percentage of CD31^+^ areas in the stained tissue within each micrograph field, shown as blood vessel coverage. Data are shown as mean ± SEM (n = 5 images from 5 mice per condition). ∗∗∗p < 0.001 (unpaired *t*-test). (C) Experimental setup for transendothelial migration of THP-1 cells or macrophage-like (Mφ) cells. (D) A monolayer of human umbilical vein endothelial cells (HUVECs) on the upper chamber of a transwell was serum-starved and then added with THP-1 or Mφ cells, either in the absence or presence of lipopolysaccharide (LPS) or N-acetyl-l-cysteine (NAC). Cells on the surface of the upper chamber opposite to the HUVEC monolayer were stained and counted. Error bars represent the mean ± SEM (n = 4 independent experiments). ∗∗p < 0.01, ∗∗∗p < 0.001 (one-way ANOVA with Bonferroni's comparison test). (E) The HUVEC monolayer, stained with Hoechst dye, was incubated with THP-1 or Mφ cells in the presence of a reactive oxygen species (ROS) dye, H_2_DCFDA (green). Fluorescence was measured at 5-min intervals using a real-time imaging setup after the addition of cells. In the micrographs, THP-1 or Mφ cells exhibit a round shape, whereas endothelial cells appear flat with the nucleus stained blue.

Another important aspect observed in the psoriasis mouse model was the abundance of blood vessels in psoriatic lesions compared to that in control mice (Fig. 2A–B). In addition, the blood vessels in the IMQ-treated skin showed increased permeability, as demonstrated by the differential accumulation of FITC-dextran in the serum, 4 h after its application to the skin of IMQ- and control-treated mice (Supplementary Fig. S3). This observation suggests that there could be a potential causal relationship between the increased number of immune cells and permeability of blood vessels, potentially through an interaction between these two cell types. To evaluate this, we established an *in vitro* co-culture model in which macrophages, differentiated from the monocyte cell line THP-1 via PMA treatment [43], were detached by trypsinization and added to a confluent monolayer of HUVECs representing blood vessels. PMA treatment to THP-1 cells indeed resulted in increased gene expression of key macrophage differentiation markers, CD11b, CD14, CD68, and TLR4 (Supplementary Figs. S4A–D), and detachment of the macrophages did not induce apoptotic death within the experimental timeline (Supplementary Fig. S4E). When this co-culture was performed in a Boyden's chamber to assess macrophage migration through the endothelial monolayer (Fig. 2C) as a measure of endothelial permeability, macrophages displayed a basal level of migration similar to that of undifferentiated THP-1 cells (Fig. 2D). However, upon treatment with the pro-inflammatory stimulus, LPS, a marked increase was observed (Fig. 2D), presumably due to the activation of its receptor TLR4 expressed on the macrophage, indicating a potential interplay between activated immune and endothelial cells. Interestingly, this increase of the macrophage migration was reduced upon treatment with the antioxidant, NAC, suggesting that the increased migration of macrophages was mediated by oxidative conditions. We repeated the migration experiment using 150 % of the number of endothelial cells required for minimum passage of FITC-dextran through the monolayer, confirmed the integrity by VE-cadherin staining, and obtained essentially the same results (Supplementary Fig. S5). The inhibitory effect of NAC on macrophage migration through the endothelial monolayer suggests that activated macrophages may increase endothelial permeability by releasing ROS, which may destabilize the endothelial monolayer. To test this hypothesis, we monitored ROS production in the co-culture setup using the cell-permeable ROS dye, H_2_DCFDA. The co-culture of THP-1 cells and HUVECs did not lead to significant changes in ROS levels, regardless of the presence of LPS (Fig. 2E, top two panels). However, the addition of LPS to the co-culture induced ROS generation in macrophages within 5 min (Fig. 2E, bottom left panel). More importantly, activated macrophages induced an increase in intracellular ROS concentrations, even within HUVECs, as evidenced 20 min after their addition (Fig. 2E, bottom panel). Although the experimental setup does not fully mimic the physiological context where macrophages typically remain within tissues rather than crossing blood vessels, these results suggest that pro-inflammatory immune cells in psoriatic lesions can induce oxidative conditions in nearby blood vessels, which may increase the vessel permeability.

### 3.3 ROS induces transendothelial migration by reducing Tie2 phosphorylation

**Fig. 3:**
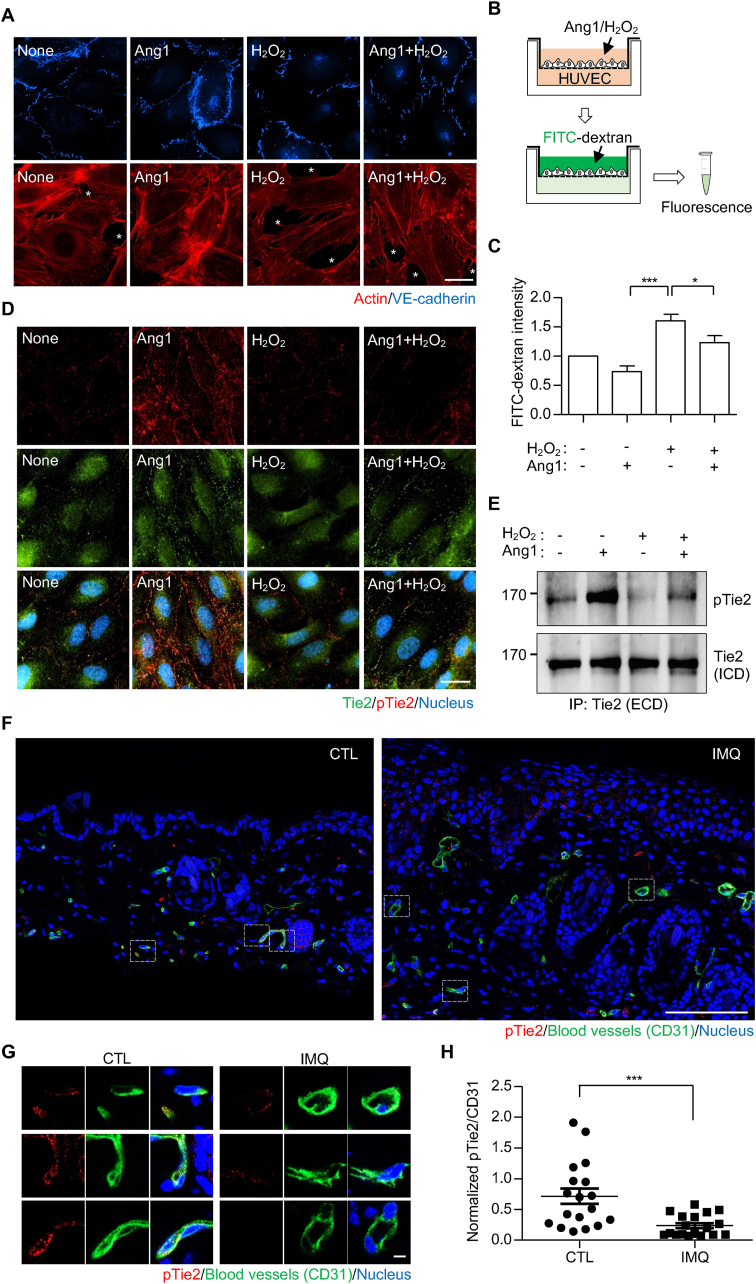
**Tie2 phosphorylation is reduced under oxidative conditions.** (A) HUVECs were starved for 6–8 h and treated with angiopoietin-1 (Ang1; 250 ng/mL) and/or H_2_O_2_ (1 mM) for 30 min, then stained with actin (red) and VE-cadherin. White asterisks indicate the empty space between endothelial cells. Scale bar, 20 μm. (B) Serum-starved HUVEC monolayer on the upper chamber of the transwell was treated with Ang1 (250 ng/mL) and/or H_2_O_2_ (1 mM) along with FITC-dextran for 30 min. (C) PBS in the lower chamber of the transwell, containing FITC-dextran that leaked from the upper chamber, was collected, and the fluorescence measured. Error bars indicate the mean ± SEM (n = 4 independent experiments). ∗p < 0.05, ∗∗∗p < 0.001 (One-way ANOVA and Bonferroni's comparison test). (D) HUVECs were starved and treated with Ang1 (250 ng/mL) and/or H_2_O_2_ (1 mM), then stained with anti-Tie2 (green), anti-phosphorylated Tie2 (pTie2; red), and Hoechst. Scale bar, 20 μm. (E) Tie2 was harvested via immunoprecipitation using an anti-Tie2 extracellular domain (ECD) antibody, and then blotted with anti-pTie2 (upper) and anti-Tie2 intracellular domain (ICD) antibodies. (F) Representative images of skin sections stained with anti-pTie2 (red), anti-CD31 (green), and Hoechst. Scale bar, 100 μm. (G) Boxed regions in (F) were digitally magnified, and images of Tie2 and CD31 staining, as well as merged images, presented. Scale bar, 5 μm. (H) Fluorescence intensities of pTie2 within CD31^+^ regions were measured and normalized against those of CD31. Dots represent CD31^+^ regions (n = 18) pooled from stained sections of 5 different mice per condition. Error bars indicate the mean ± SEM. ∗∗∗p < 0.001 (unpaired *t*-test).

The correlation between the degree of transendothelial migration and ROS concentrations in HUVECs suggests that increased ROS concentrations may regulate junctional stability of the endothelial layer. To test this, we treated HUVECs with H_2_O_2_, a major ROS, and observed endothelial cell junctions by staining for VE-cadherin. Under normal conditions, HUVECs appeared to be connected via VE-cadherin at their points of contact with neighboring cells. Upon treatment with angiopoietin-1 (Ang1), an agonistic ligand of the vessel-stabilizing Tie2 receptor [44,45], cell-cell contacts were more tightly sealed, as observed by actin staining, leaving limited spaces between cells (Fig. 3A). In contrast, treatment with H_2_O_2_ disrupted the junction, creating more space between the HUVECs (Fig. 3A, white asterisks). Treatment of cells with 1 mM H_2_O_2_ also caused a significant increase in F-actin to G-actin ratio, suggesting changes in cytoskeleton architecture during this process (Supplementary Figs. S6A and S6B). Consistently, in the Boyden's chamber (Fig. 3B), the leakage of FITC-dextran from the upper to the lower chamber through the HUVECs monolayer was significantly increased after H_2_O_2_ treatment (Fig. 3C and Supplementary Fig. S6C), indicating that ROS can likely increase the permeability of endothelial cells. Notably, simultaneous treatment with Ang1 partially but significantly reversed the H_2_O_2_-mediated increase in vessel permeability (Fig. 3A and C), indicating the potential involvement of the Ang1-Tie2 signaling axis in the response of endothelial cells to ROS. The partial reversal seems to result from H_2_O_2_'s effects on multiple factors involved in vascular permeability, including reductions in the basal phosphorylation of VE-cadherin and Tie1 (Supplementary Figs. S7A and S7B). In addition, although the direct reduction of VE-cadherin phosphorylation by H_2_O_2_ made precise assessment challenging, the VE-PTP-mediated additive effect on VE-cadherin dephosphorylation does not appear to be affected by H_2_O_2_ (Supplementary Fig. S7A, lane 4 vs 6), suggesting that VE-PTP activity remains unchanged under the condition. Based on the reversal effect of Ang1 in H_2_O_2_-induced permeability (Fig. 3C), we focused on Tie2 and examined Tie2 activation in this process by measuring the degree of phosphorylation at its auto-phosphorylation site, Tyr992 [46]. Interestingly, H_2_O_2_ treatment largely inhibited both basal and Ang1-induced Tie2 phosphorylation, as observed in Tie2 staining (Fig. 3D) and immunoprecipitation (Fig. 3E) in HUVECs. Moreover, the co-culture of HUVECs with LPS-stimulated macrophages reduced Ang1-induced Tie2 phosphorylation, which was restored by co-treatment with the antioxidant NAC (Supplementary Fig. S7C), suggesting that physiological secretion of ROS from immune cells can also inhibit Tie2 phosphorylation. Furthermore, not only its activation, but also its activity, measured by its ability to induce Tie1 phosphorylation, was suppressed by H_2_O_2_ (Supplementary Fig. S7B, lane 4 and 7). Collectively, these results suggest that ROS from immune cells can potentially induce blood vessel permeability by reducing Tie2 phosphorylation. If this is the case, an increased oxidative environment in psoriatic lesions (Fig. 1I) might reduce Tie2 phosphorylation, thereby increasing vessel permeability and facilitating the enhanced recruitment of immune cells to the lesion (Fig. 1F). To test this hypothesis, we stained blood vessels in skin sections from our psoriasis mouse model using anti-CD31 and anti-phosphorylated Tie2 antibodies (Fig. 3F). We then focused on the CD31-positive regions, measured the fluorescence intensities of both CD31 and phosphorylated Tie2 staining (Fig. 3G), and calculated the ratio of the intensities in both control and IMQ-treated mice. In this way, we confirmed a significant reduction in the levels of phosphorylated Tie2 in psoriasis (Fig. 3H). Co-immunostaining using anti-Tie2 and anti-CD31 antibodies showed that there is no obvious difference in Tie2 expression (Supplementary Fig. S8).

### 3.4 Tie2 kinase domain can serve as a direct target of ROS

**Fig. 4:**
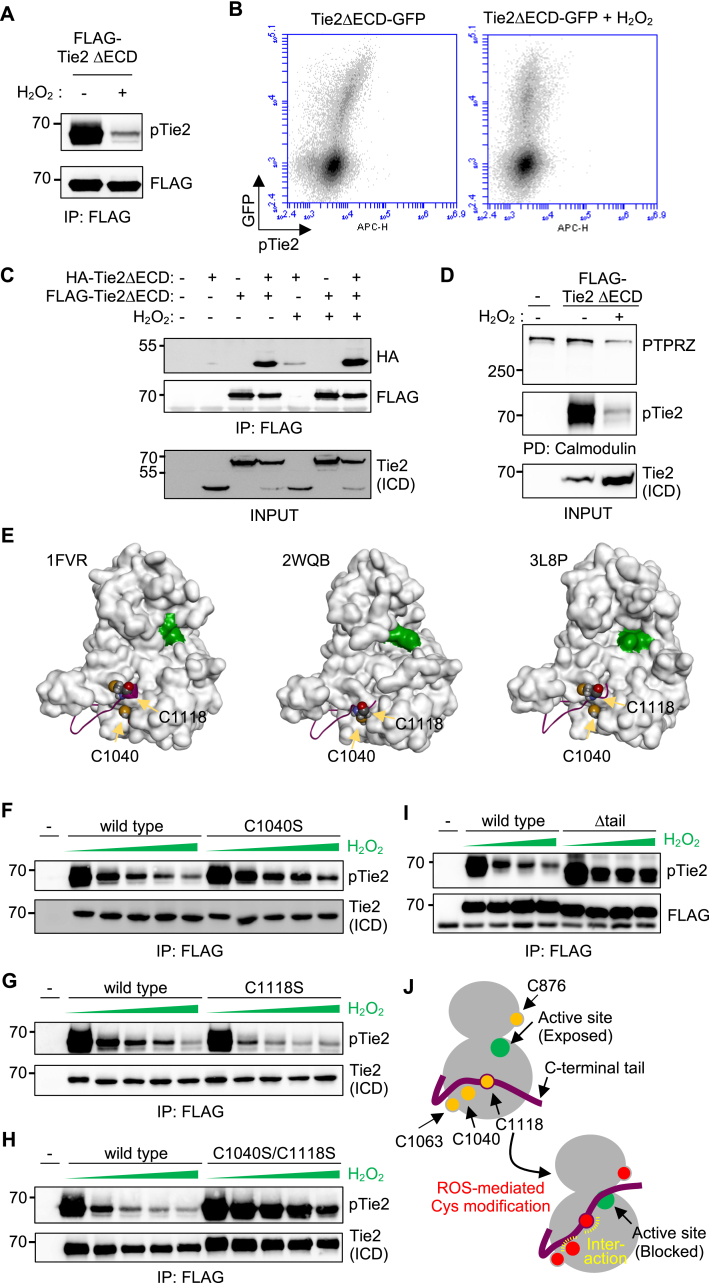
**Cysteines in the Tie2 kinase domain are crucial for the ROS-mediated decrease in Tie2 phosphorylation.** (A) FLAG-tagged Tie2ΔECD transfected into HEK293T cells was precipitated using anti-FLAG antibody and blotted using anti-pTie2 and anti-FLAG antibodies. (B) HEK293T cells expressing Tie2ΔECD fused with GFP were starved and treated with 1 mM H_2_O_2_ for 30 min. The resulting cells were analyzed using intracellular staining of pTie2 and flow cytometry. (C) HEK293T cells were transiently transfected with Tie2ΔECD tagged with HA and/or FLAG. The cells were then serum-starved and treated with or without H_2_O_2_ (1 mM) for 30 min. FLAG-Tie2ΔECD was immunoprecipitated, and the bound HA-Tie2ΔECD analyzed via western blotting (upper). Existence of these two versions of Tie2ΔECD was verified via an anti-Tie2 ICD antibody (bottom). (D) The cell lysates of HEK293T cells transfected with FLAG-Tie2ΔECD and treated with H_2_O_2_ (1 mM) were incubated with calmodulin-beads, and the bound PTPRZ (upper), pTie2 (middle), and total Tie2 (bottom) analyzed via western blotting. (E) The Tie2 kinase domain surface structure from the Protein Data Bank (PDB ID: 1FVR, 2WQB, 3L8P) is depicted, with the Asp-Phe-Gly motif within the kinase active site highlighted in green. To enhance clarity, certain residues have been hidden in the surface image. The C-terminal tail, from residues 1102–1124, is represented in a ribbon model and colored magenta. Note that the region spanning from residues 1121–1124 is not visible in the structure due to structural fluctuation. Cys1040 and Cys1118 are presented in a space-filling model. (F, G, H, I) HEK293T cells were transfected with either FLAG-Tie2ΔECD or its mutants, including C1040S, C1118S, the double mutant (C1040S/C1118S), or the C-terminal tail-deleted mutant (Δtail; deletion from 1102 to 1124). Phosphorylated Tie2 was analyzed via immunoprecipitation. These experiments were performed independently more than three times with similar results. (J) Proposed mechanism of how the C1040 and C1118 are involved in ROS-mediated Tie2 inactivation. H_2_O_2_ concentration in (F–H), 0, 0.3, 0.4, 0.45, 0.5 mM; in (I), 0, 0.3, 0.4, 0.5 mM.

We investigated the mechanism by which H_2_O_2_ reduces Tie2 phosphorylation. A Tie2 mutant lacking the extracellular domain (Tie2ΔECD) is constitutively active, possibly due to the absence of an inhibitory element in the extracellular domain affecting the intrinsic oligomerization properties of Tie2 [47]. Consequently, overexpression of this mutant in any cell type, such as HEK293T cells, typically induced phosphorylation of Tie2, even in the absence of its ligand (Fig. 4A, lane 1). Interestingly, H_2_O_2_ treatment resulted in a marked reduction in the observed phosphorylation (Fig. 4A, lane 2). The decreased phosphorylation of Tie2ΔECD fused with GFP was also confirmed via intracellular flow cytometry (Fig. 4B). The decreased phosphorylation was also induced by a different oxidizing reagent, KBrO_3_ (Supplementary Fig. S9), which likely induces an oxidative environment during its conversion into Br^−^ [48]. These results indicate that the extracellular domain of Tie2 or its binding to Ang1 is not a direct target of H_2_O_2_, and that the effect of H_2_O_2_ was not specific to endothelial cells, as decreased phosphorylation occurred in non-endothelial HEK293T cells. We next investigated whether H_2_O_2_ affects the oligomerization of Tie2ΔECD. When the interaction of differently tagged Tie2DECD, HA-Tie2DECD, and FLAG-Tie2DECD were assessed via immunoprecipitation, no significant changes induced by H_2_O_2_ were observed (Fig. 4C). Previously, we discovered a potential role for PTPRZ, a member of the receptor-type protein tyrosine phosphatase family, in Tie2 dephosphorylation when both proteins are sequestered by calmodulin [35]. H_2_O_2_ treatment, however, did not affect the calmodulin-dependent interaction between Tie2 and PTPRZ (Fig. 4D), suggesting that this interaction was also not the target. Given that H_2_O_2_ reduces Tie2 phosphorylation even in the absence of the extracellular domain in a cell type-independent manner and is effective on overexpressed Tie2, it potentially surpasses any H_2_O_2_-dependent cellular machinery responsible for Tie2 inactivation. We thus hypothesized that the Tie2 kinase domain might directly react with H_2_O_2_, leading to its reduced phosphorylation.

### 3.5 ROS-mediated modification of cysteines in the Tie2 kinase domain can reduce its phosphorylation

To test this hypothesis, we thoroughly examined the available structural information on the Tie2 kinase domain from the public RCSB Protein Data Bank database (Fig. 4E). As cysteine residues can be influenced by oxidative stress, either by forming disulfide bonds or by being modified [49], we focused our investigation on cysteine residues within the Tie2 kinase domain. Interestingly, in one of the Tie2 kinase domain structures, we observed a disulfide bond between Cys1040 and Cys1118, which potentially stabilizes the position of the C-terminal tail near the active site (Fig. 4E; 2WQB). As the C-terminal tail functions to inhibit kinase activity by blocking entry of the substrate into the active site [46], stabilization of its position through disulfide bond formation would likely inhibit Tie2 activity. Therefore, we hypothesized that disulfide bond formation between these two cysteine residues under oxidative conditions may explain the observed decrease in Tie2 phosphorylation. However, the Tie2ΔECD mutant introduced either with Cys1040Ser or Cys1118Ser, which would sufficiently destroy the predicted disulfide bond formation, remained sensitive to H_2_O_2_ exposure (Fig. 4F–G). Nonetheless, the mutation of both cysteine residues to serine largely desensitized the mutant (Fig. 4H). This suggests that the intact presence of one of the cysteine residues is essential for the H_2_O_2_-mediated decrease in Tie2 phosphorylation, possibly through their modification, leading to Tie2 adopting an inactive conformation rather than forming a disulfide bond. Notably, when the inhibitory C-terminal tail was deleted, the decrease in Tie2 phosphorylation was largely inhibited (Fig. 4I), suggesting its importance in H_2_O_2_-mediated Tie2 regulation. In addition, mass spectrometry analysis of the trypsinized fragments of Tie2ΔECD purified from ROS-treated HEK293T cells revealed that, although the entire sequence, including the region containing Cys1040 and Cys1118, was not covered (Supplementary Fig. S10A), other cysteines, Cys876 and Cys1063, can undergo triple oxidation (Supplementary Fig. S10B). Consistently, substitution of these residues with acidic Glu reduced Tie2 phosphorylation even without H_2_O_2_, to varying degrees for each cysteine (Supplementary Fig. S10C). Taken together, these results suggest that ROS can modify cysteines at multiple sites in the Tie2 kinase domain with different sensitivity to H_2_O_2_ and different impacts on Tie2 activation, potentially shifting the equilibrium gradually toward an inactive conformation depending on the extent of such modifications, for example, by repositioning the inhibitory C-terminal tail near the active site (Fig. 4J).

### 3.6 Activating Tie2 can overcome endothelial dysfunction under oxidative conditions

**Fig. 5:**
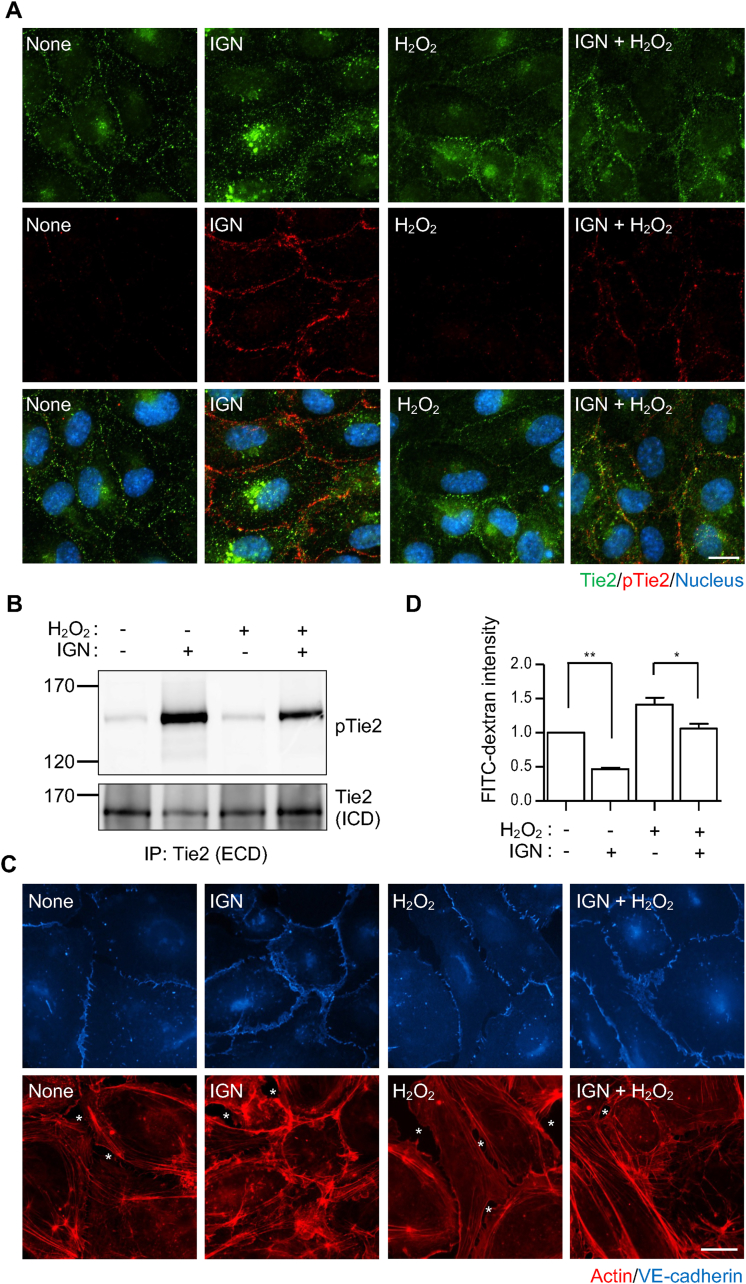
**Activating Tie2 can overcome the ROS-mediated increase in endothelial permeability.** (A, B, C) HUVECs were starved and treated with IGN-002 (20 μg/mL) and/or 1 mM H_2_O_2_ for 30 min. Subsequently, the cells were either stained with anti-Tie2 (green), anti-pTie2 (red), and Hoechst (A), subjected to immunoprecipitation as in Fig. 3E (B), or stained with VE-cadherin (blue) and actin (red) (C). White asterisks indicate empty spaces between endothelial cells. Scale bar, 20 μm. (D) The effect of IGN-002 on endothelial permeability was analyzed as in Fig. 3B and C. Error bars represent the mean ± SEM (n = 4 independent experiments). ∗p < 0.05, ∗∗p < 0.01 (one-way ANOVA with Bonferroni's comparison test). Scale bar, 20 μm.

As the reduced phosphorylation of Tie2 can cause vessel permeability in an oxidative environment, we attempted to reverse this effect by reactivating Tie2 using the Tie2-activating antibody, IGN-002, which could potentially shift the equilibrium toward Tie2 activation even under Tie2-inhibiting oxidative conditions. As observed with Ang1 treatment (Fig. 3A), treatment of HUVECs with IGN-002 resulted in elevated levels of Tie2 phosphorylation under both normal and H_2_O_2_-treated conditions compared to those without IGN-002 treatment (Fig. 5A). In the immunoprecipitation assay, IGN-002 treatment also reversed the H_2_O_2_-induced reduction in Tie2 phosphorylation (Fig. 5B). Moreover, IGN-002 effectively sealed the cell-cell contacts between HUVECs, even in the presence of H_2_O_2_, thus reducing gaps within the monolayer (Fig. 5C). Consistently, H_2_O_2_-induced endothelial permeability, as assessed via the passage of FITC-dextran through the endothelial monolayer in the Boyden's chamber, was significantly reduced (Fig. 5D). Based on these results, we concluded that inducing Tie2 activation can reduce ROS-mediated endothelial dysfunction.

**Fig. 6:**
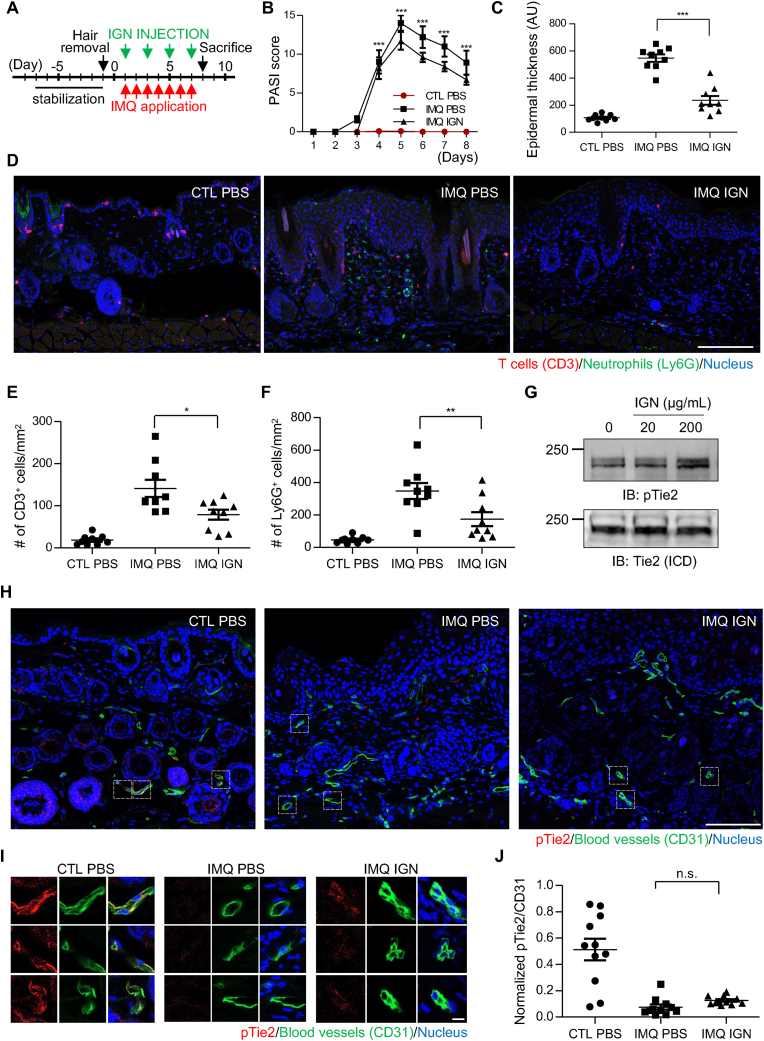
**Tie2-activating antibody alleviates the severity of psoriasis in a mouse model.** (A) IGN-002 was subcutaneously injected into IMQ-treated mice at 10 mg/kg every two days for a duration of seven days. (B) PASI scores of CTL-treated and PBS-injected (CTL PBS), IMQ-treated and PBS-injected (IMQ PBS), and IMQ-treated and IGN-002-injected (IMQ IGN) mice are presented as in Fig. 1c. Error bars represent the mean ± SEM (n = 3 mice). ∗∗∗p < 0.001 (two-way ANOVA with Bonferroni's comparison test). (C) Epidermal thickness of these three groups of mice was analyzed as in Fig. 1E n = 9 samples from 3 mice per condition. ∗∗∗p < 0.001 (one-way ANOVA with Bonferroni's comparison test). (D, E, F). Skin sections from these three groups of mice were stained with anti-CD3 (red), anti-Ly6G (green), and Hoechst, and analyzed as in Fig. 1F, G, and 1H. Error bars represent the mean ± SEM (n = 9 samples from 3 mice per condition). ∗p < 0.05, ∗∗p < 0.01 (one-way ANOVA with Bonferroni's comparison test). (G) CHO cells expressing mouse Tie2 fused with GFP were treated with IGN-002 at the indicated concentration for 30 min, and the degree of Tie2 phosphorylation analyzed via western blotting using an anti-pTie2 antibody. (H-J) Skin sections of mice from the three groups were stained with anti-pTie2, anti-CD31, and Hoechst, and analyzed as in Fig. 3F, G, and 3H n = 11 CD31^+^ regions pooled from stained section of 3 mice per condition. n.s., not significant (one-way ANOVA with Bonferroni's comparison test).

Finally, we investigated whether Tie2 activation could potentially alleviate the increased inflammation in psoriatic lesions. We administered IGN-002 every two days via intraperitoneal injection at a dose of 10 mg/kg to control or IMQ-treated mice (Fig. 6A) and conducted various pathological examinations. According to the blinded PASI scores, IGN-002-injected mice exhibited a mild benefit compared to that in the control group (Supplementary Figure S11 and Fig. 6B). Hematoxylin and eosin-stained sections from day 8 mice (Supplementary Fig. S12) also indicated a reduction in epidermal thickness following IGN-002 injection (Fig. 6C). Additionally, the numbers of T cells and neutrophils in the lesions were significantly decreased in IGN-002-treated mice (Fig. 6D–F). However, their levels remained still higher than those in the control group, potentially explaining its limited benefit observed in the PASI scores. Thus, none of these indices were lower than expected; they did not restore the defect observed in IMQ-treated mice to the level observed in control mice without IMQ treatment. Unfortunately, although IGN-002 proved to be highly effective in activating human Tie2 expressed in HUVECs (Fig. 5A–B), its efficacy was not robustly replicated in mouse Tie2 that was stably expressed in cell lines (Fig. 6G) or mouse skin tissue (Fig. 6H–J). This discrepancy appears to stem from the variation in the 707th residue of Tie2 within the IGN-002 epitope [50], which is valine in humans but arginine in mice. Although maximal Tie2 activation was not achieved due to the species specificity of the antibody used, the reduced PASI score and immune cell infiltration following IGN-002 administration suggest that Tie2 activation can be used as a therapeutic strategy for psoriasis.

## 4 Discussion

In our mouse model of psoriasis, we observed a significant increase in oxidative stress markers, likely due to the elevated number of T cells and neutrophils within the psoriatic lesions (Fig. 1). Additionally, oxidative conditions were evident within the pro-inflammatory immune cells in our *in vitro* co-culture model, in which these oxidative conditions were transmitted to endothelial cells (Fig. 2). We demonstrated that H_2_O_2_ treatment, which mimics oxidative conditions, could impair the junctional stability of endothelial cells, thus facilitating the transendothelial migration of immune cells (Fig. 2). This may explain the prolonged maintenance of inflammation through a vicious cycle of immune cell infiltration, followed by the induction of leaky vessels by these immune cells, thereby promoting further infiltration in psoriasis. Importantly, we discovered that H_2_O_2_-mediated vessel destabilization was, at least in part, due to its role in inactivating Tie2 (Fig. 3), possibly through the modification of cysteines in the kinase domain of Tie2 (Fig. 4). Based on these findings, we propose that activating Tie2 in psoriasis may counteract vessel destabilization and immune cell infiltration (Fig. 5), ultimately reducing the inflammatory condition in psoriasis (Fig. 6) by stopping the vicious cycle.

Tie2, a receptor tyrosine kinase that is predominantly expressed in endothelial cells, plays a crucial role in vascular stability. Activation of Tie2 signaling by its agonistic ligand, Ang1, is known to stabilize blood vessels by reinforcing endothelial cell-cell contacts, thereby reducing vessel permeability [51,52]. Conversely, the antagonistic ligand of Tie2, Ang2, disrupts vessel stability, leading to increased vessel permeability and excessive immune cell infiltration [53]. Owing to the stabilizing role of Tie2, the activation of Tie2, for example, by inhibiting vascular endothelial-protein tyrosine phosphatase, a phosphatase responsible for Tie2 dephosphorylation, can prevent an increase in vessel permeability and immune cell extravasation [54]. This Tie2-mediated vessel stabilization also has significant implications for cancer treatment because abnormal blood vessel growth induced by cancer cells leads to the formation of leaky vessels, facilitating cancer cell dissemination and metastasis [55]. Additionally, these abnormal vessels impair the proper delivery of chemotherapeutic drugs to the cancerous region [56]. Therefore, promoting blood vessel stabilization, also known as vessel normalization, emerged as a promising therapeutic strategy for cancer treatment [57,58]. Indeed, various approaches have been explored to modulate the Ang-Tie2 signaling pathway in cancer therapy, including the use of antibodies targeting Ang1 or Ang2 [59]. However, our findings suggest that Tie2 in endothelial cells is not solely regulated by its ligands. Instead, its activation state is also influenced by interactions with immune cells through the oxidative conditions these cells create or induce, as well as possibly by interactions with cancer cells that are known to have elevated ROS levels [60]. Thus, we suggest that therapeutic strategies targeting Tie2 signaling for enhanced vessel stability should consider the intricate interplay between endothelial cells and their neighbors.

A pioneering study demonstrated that H_2_O_2_ can increase phosphorylation of the receptor tyrosine kinase, platelet-derived growth factor receptor [61]. It was later discovered that H_2_O_2_ can induce oxidation of the catalytic cysteine residue within protein tyrosine phosphatases, thereby inhibiting their activity [62]. Consequently, this inhibition favors the increased phosphorylation of their target proteins, including the platelet-derived growth factor receptor. In contrast, we observed a decrease in Tie2 phosphorylation after H_2_O_2_ exposure. In the case of Tie2, H_2_O_2_ may directly induce oxidation of the sulfhydryl groups of cysteines, including Cys1040 and Cys1118 (Fig. 4H), possibly converting them into sulfenic acid (-SOH) or further oxidized chemicals [63]. Indeed, our mass analysis identified triple oxidation of Cys876 and Cys1063, which results in conversion of thiols into sulfonic acids (Supplement Fig. 10). Thus, we hypothesize that the acquired negative charge, or oxygen itself, on the oxidized cysteines may interact with their neighbors via electrostatic interactions or hydrogen bonds, favoring conformation of the inactive structure (Fig. 4J). Therefore, Tie2 could function as a ROS sensor, with its activity modulated depending on the environmental redox conditions; although the precise nature of cysteine modification requires further investigation. We also note that external activation signals, such as an agonist (Fig. 3C) or an activating antibody (Fig. 5B), can restore the equilibrium.

Then, how can immune cells induce the increase of H_2_O_2_ in endothelial cells, as observed in our results (Fig. 2E)? One possible mechanism is that H_2_O_2_ released from immune cells can be uptaken by endothelial cells through aquaporins, water channels, which are also known to transport external H_2_O_2_ into cells [64]. Alternatively, differentiated THP-1 cells, or macrophages, may produce signaling molecules, such as TNF-alpha, upon LPS exposure [65], which can stimulate HUVECs to produce ROS [66]. Exploring these hypothesis will be an interesting topic for future studies, which can provide additional strategies for blocking inflammatory diseases.

In conclusion, our study suggests that activating Tie2 could offer an alternative therapeutic approach for treating psoriasis, particularly in patients with various immunological disorders or those experiencing adverse effects from conventional drugs. Additionally, our findings indicate that Tie2 may serve as a direct receptor for sensing oxidative conditions. Further investigation into the role of Tie2 in these contexts could contribute to the development of advanced treatment strategies not only for psoriasis, but also for other conditions characterized by compromised blood vessel integrity, such as inflammation and cancer.

## CRediT authorship contribution statement

**An Vuong Quynh Pham:** Writing – review & editing, Writing – original draft, Investigation, Data curation. **Yongwoo Na:** Writing – review & editing, Validation, Data curation. **Gyeongseo Suk:** Writing – review & editing, Data curation. **Chansik Yang:** Writing – original draft, Conceptualization. **So Min Kang:** Writing – original draft, Validation, Data curation. **Joonha Lee:** Formal analysis. **Hongseo Choi:** Investigation. **Wook Kim:** Formal analysis, Investigation. **Sung Wook Chi:** Investigation, Resources. **Sangyeul Han:** Writing – review & editing, Writing – original draft, Validation, Data curation. **Hae Woong Choi:** Writing – review & editing, Data curation. **Hyeonwoo Kim:** Writing – review & editing, Validation, Data curation. **Chungho Kim:** Writing – review & editing, Writing – original draft, Supervision, Project administration, Investigation, Funding acquisition, Formal analysis, Data curation.

## Data availability

Data are available in the main text or the supplementary materials. The original LC-MS/MS datasets and related identification files used to support this paper have been deposited to the Proteome Xchange Consortium via the PRIDE partner repository with the dataset identifier, <PXD058810>.

## Declaration of competing interest

The authors declare the following financial interests/personal relationships which may be considered as potential competing interests: No conflict of interest exists except with S. Han, who is an employee and a stockholder of Ingenia Therapeutics Inc.
