# Supplementary material for: Identification of Tie2 as a sensor for reactive oxygen species and its therapeutic implication
Source: Redox Biol. 2025 Feb 20;81:103555. doi: 10.1016/j.redox.2025.103555 (PMC11903958; doi:10.1016/j.redox.2025.103555)
Supplement: Multimedia component 1 [file mmc1.pdf]

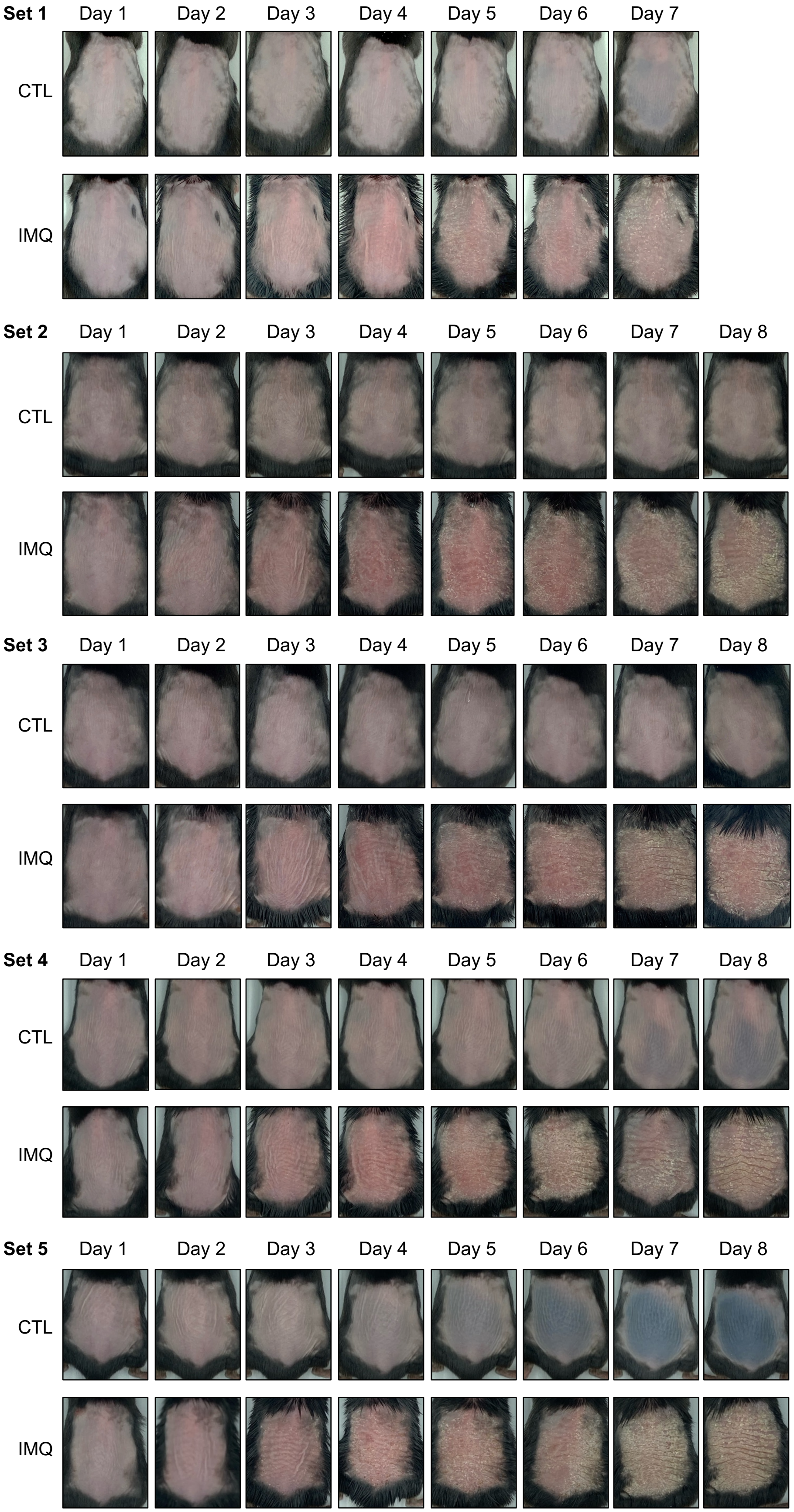

Supplementary Figure S1. Back skin images of five mouse groups treated with control cream (CTL) or imiquimod (IMQ) for seven days. Images were taken right before cream application.

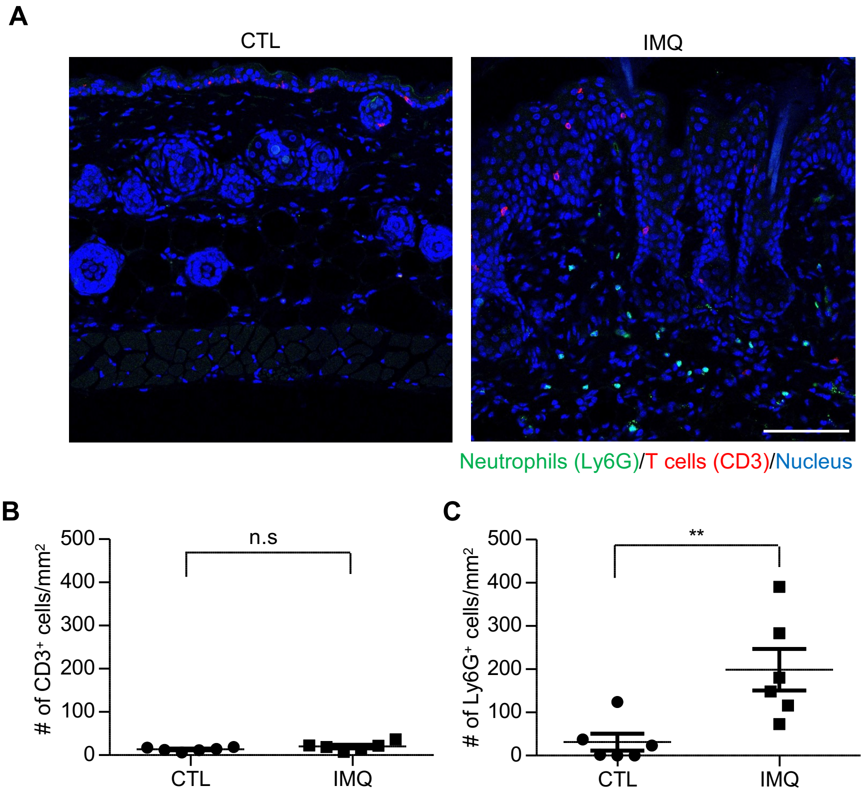

Supplementary Figure S2. (A) Skin sections of mice treated with either CTL or IMQ for five days were stained with anti-CD3 (red), anti-Ly6G (green), and Hoechst (blue). (B, C) The number of cells were quantified as in Figure 1G and 1H. Error bars represent the mean ± SEM (n = 6 samples from 3 mice per condition). ** $p < 0.01$ (unpaired t-test). n.s., not significant. Scale bar, 100 μm.

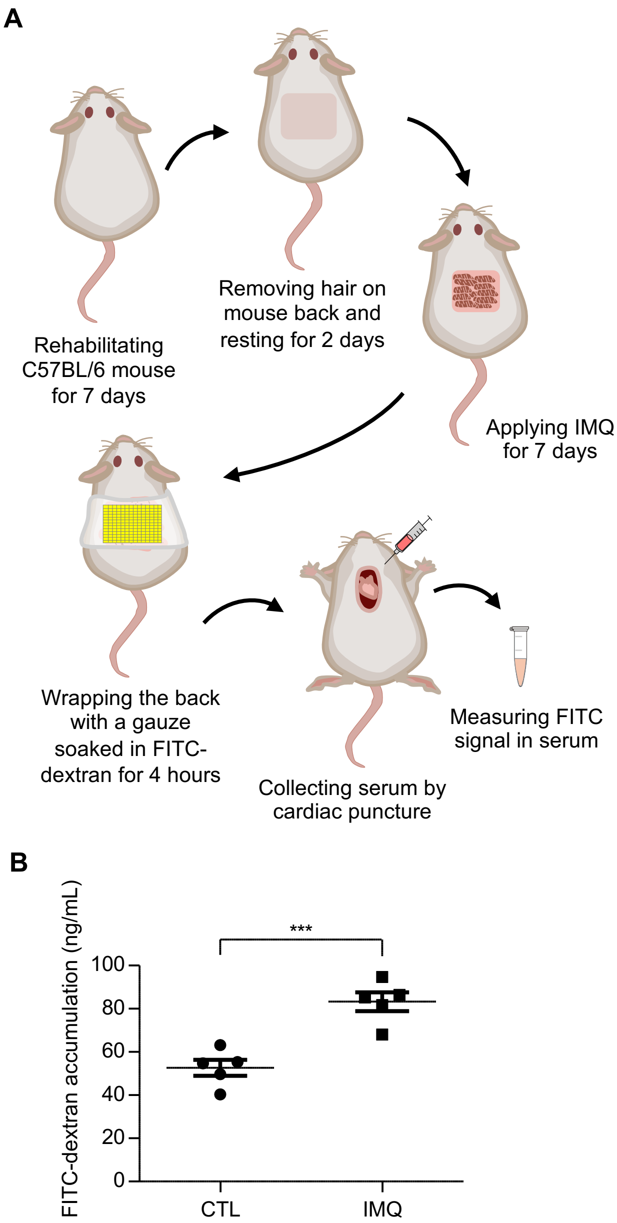

Supplementary Figure S3. (A) Experimental scheme for *in vivo* permeability assay in mouse. Back skin of mice treated for seven days with either control cream (CTL) or imiquimod (IMQ) was covered with gauze soaked in 1 mg FITC-dextran dissolved in 1 ml PBS. The amount of FITC-dextran absorbed into the blood after 4 hours of application was measured using a fluorometer. (B) Quantification of FITC-dextran accumulation in mouse serum. Error bars represent the mean ± SEM (n = 5 mice). *** $p < 0.001$ (unpaired t-test).

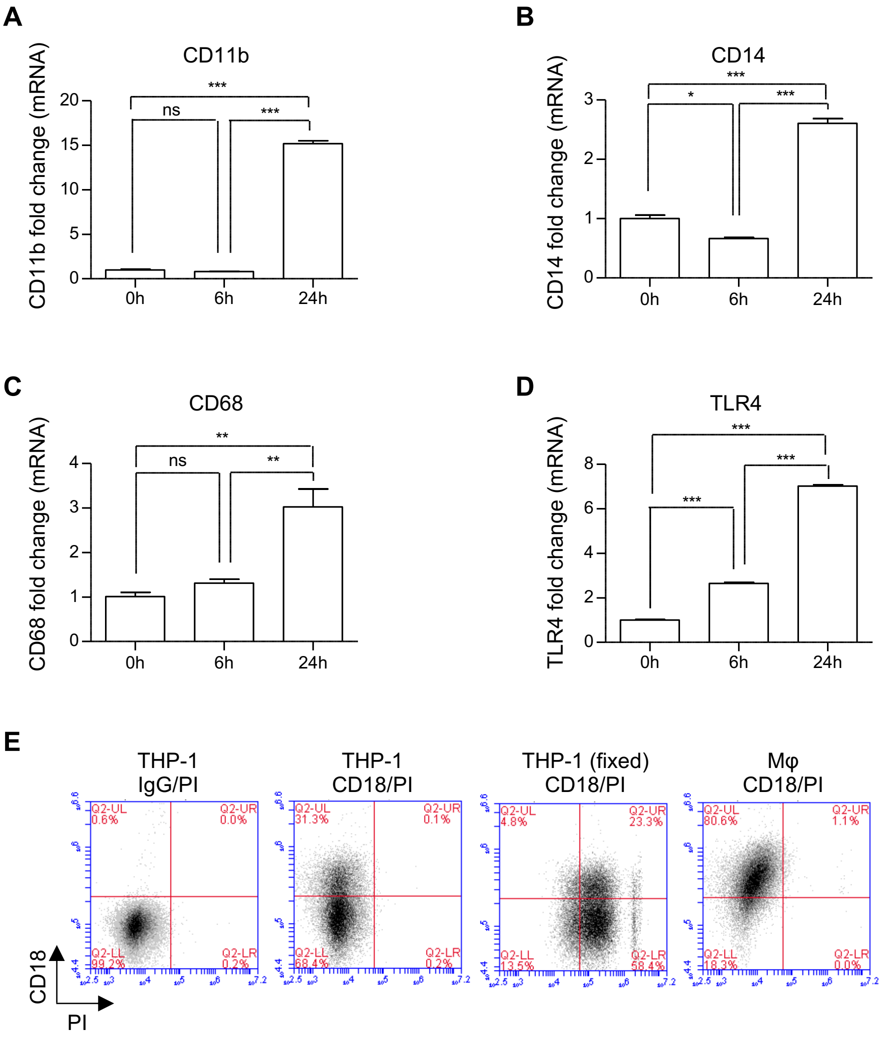

Supplementary Figure S4. Quantification of (A) CD11b, (B) CD14, (C) CD68, and (D) TLR4 mRNA expression in THP-1 after 0, 6 or 24 hours of treatment with PMA. Data are shown as mean ± SEM (n = 3 independent experiments). Data were analyzed using one-way ANOVA with Bonferroni's comparison test. ** $p < 0.01$, *** $p < 0.001$. (E) THP-1 cells were stained with control IgG or anti-CD18, followed by incubation with propidium iodide (PI) for 10 minutes. As a positive control for PI staining, CD18-stained THP-1 cells were fixed with formaldehyde before PI addition. Macrophage-like (Mφ) cells, differentiated from THP-1 by PMA treatment for 48 hours, were detached by trypsinization and stained with anti-CD18 and PI. Flow cytometry analysis, shown as dot plots, revealed negligible PI-positive cells in macrophages.

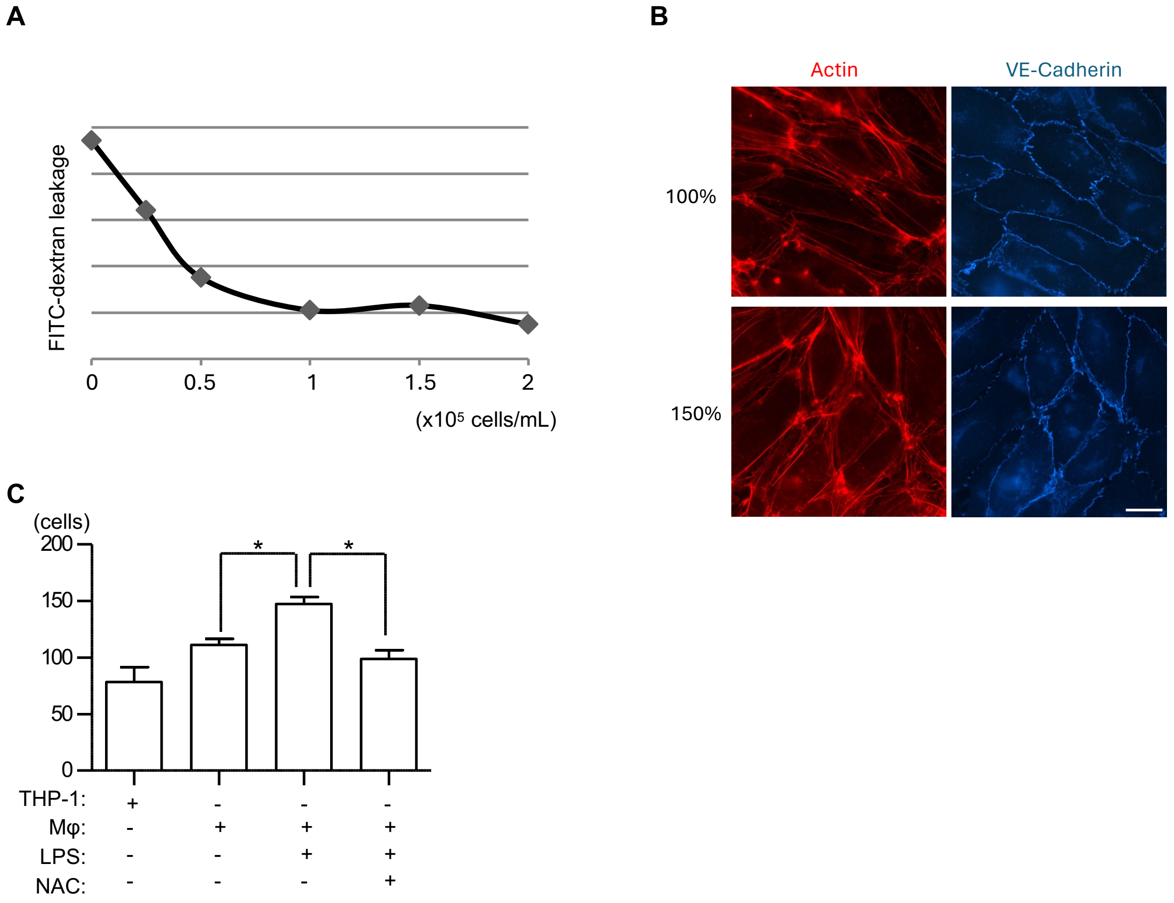

Supplementary Figure S5. (A) HUVECs were incubated for 24 hours on the upper chambers of transwell at different seeding density as indicated, and the permeability to FITC-dextran was measured as in Figure 3C. (B) HUVECs at 100% confluency (seeding density: 1.0 x 10^5 cells/mL) and 150% confluency (seeding density: 1.0 x 10^5 cells/mL) were stained for actin (red) and VE-cadherin (blue) as in Figure 3A. Scale bar, 20 μm. (C) Transendothelial migration assay was performed using 150% confluent HUVEC monolayer as in Figure 3C. Error bars indicate the mean ± SEM (n = 3 independent experiments). *, $p < 0.05$ (one-way ANOVA with Bonferroni's comparison test).

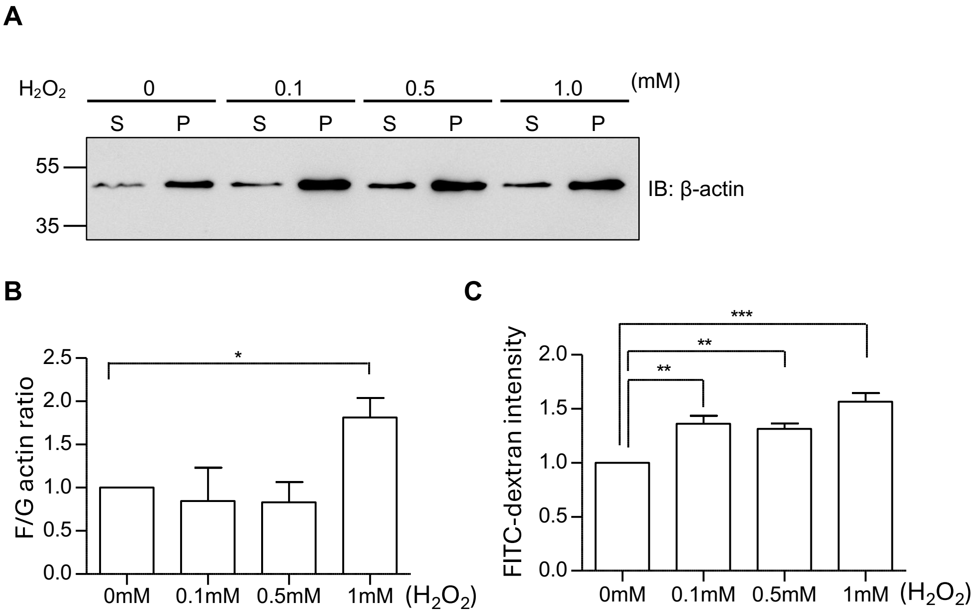

Supplementary Figure S6. (A) HUVECs were starved then treated with various concentrations of H_2O_2 as indicated for 30 minutes. Cell lysates were centrifuged at 16,000 g, and the resulting supernatants and precipitates were analyzed with western blot using anti β-actin antibody. (B) The ratio of F-actin (precipitate) to G-actin (in supernatant) was normalized to 0 mM H_2O_2 condition for each experiment and shown as bar graph. Error bars indicate the mean ± SEM (n = 3). * $p < 0.05$ (one-way ANOVA with Bonferroni's comparison test). (C) Confluent monolayer of HUVECs was treated with various amount of H_2O_2 for 30 minutes as indicated, the permeability to FITC-dextran was measured as in Figure 3C. Data are shown as mean ± SEM (n = 3 independent experiments), ** $p < 0.01$, *** $p < 0.001$ (one-way ANOVA with Bonferroni's comparison test).

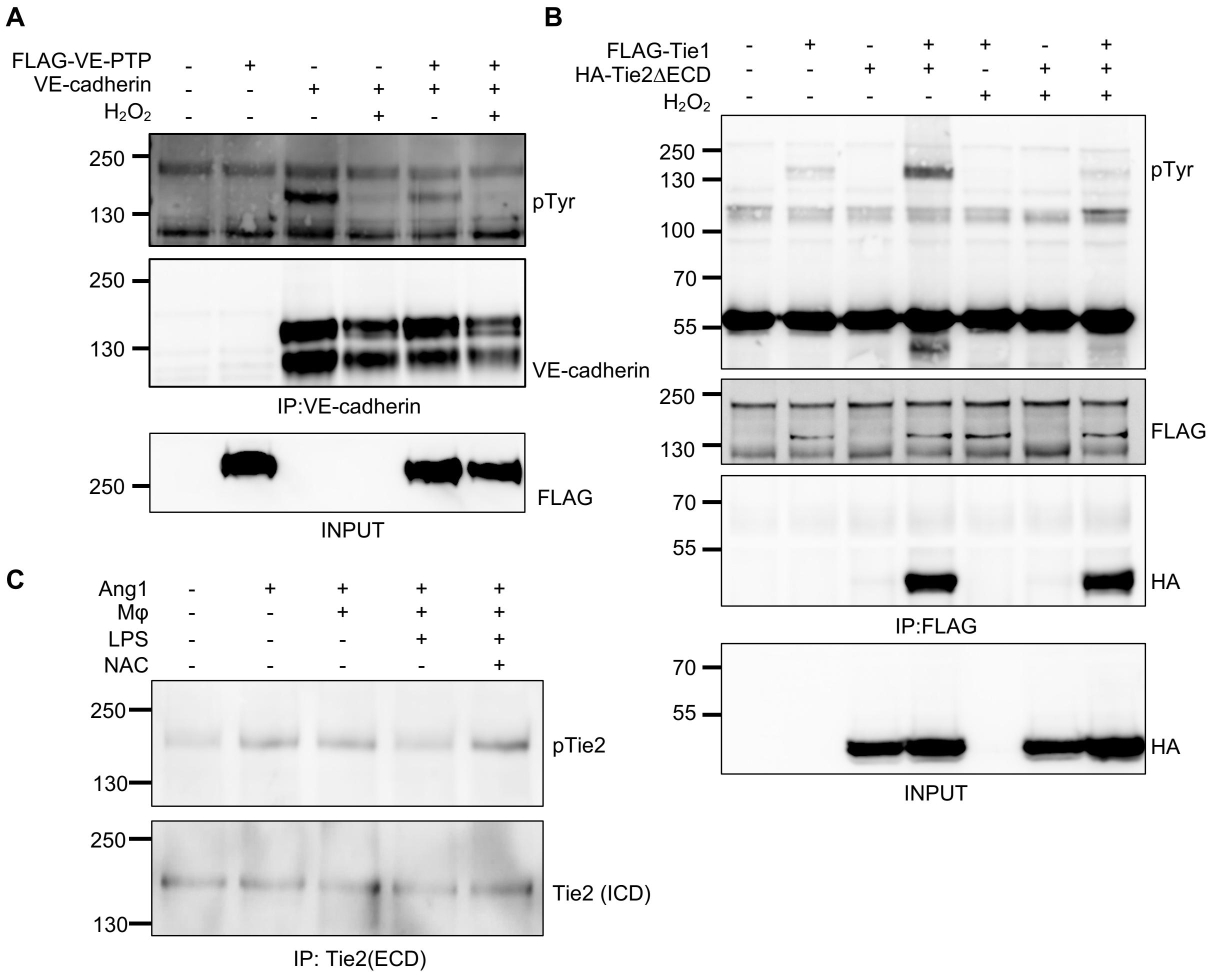

Supplementary Figure S7. (A) HEK293T cells were transfected with cDNAs encoding FLAG-tagged VE-PTP and VE-cadherin, starved for serum, and treated with 1 mM H_2O_2 for 30 minutes. VE-cadherin in the cell lysates were precipitated and analyzed with western blot using anti-phosphotyrosine (pTyr) and anti-VE-cadherin antibodies. (B) HEK293T cells transfected with FLAG-tagged Tie1 and HA-tagged Tie2ΔECD were serum-starved, and treated with 1 mM H_2O_2 for 30 minutes. Anti-FLAG immunoprecipitates were analyzed by western blot using anti-phosphotyrosine, anti-FLAG, and anti-HA antibodies. (C) HUVEC monolayers were serum-starved for 8 hours before co-culture with immune cells in different conditions. Co-culture were maintained for 1 hour, with or without Ang1 (250 ng/mL) added during the final 30 minutes. Tie2 phosphorylation was analyzed as in Figure 3E.

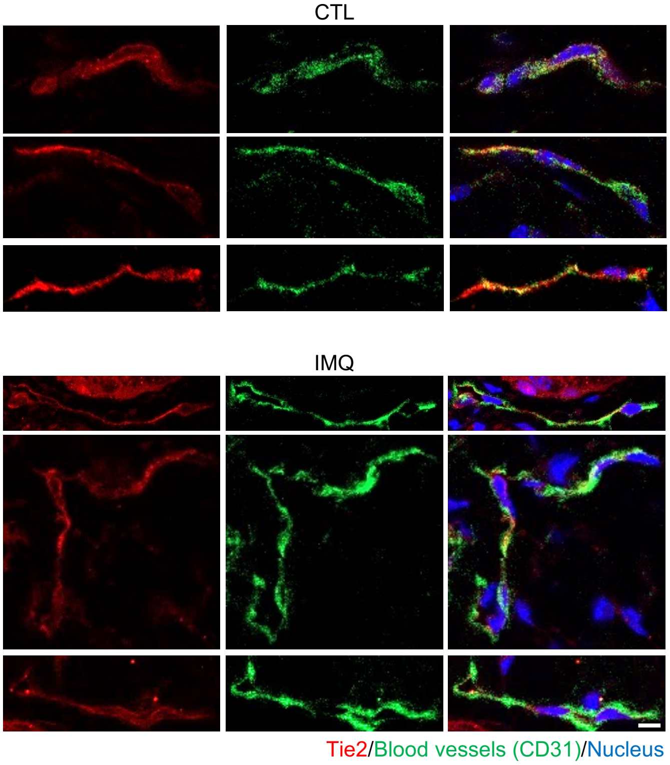

Supplementary Figure S8. Representative of image of control or IMQ-treated skin section stained with Tie2 (red) and CD31 (green). Scale bar, 5 μm.

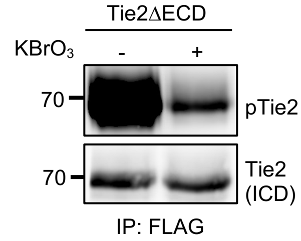

Supplementary Figure S9. HEK293T cells expressing FLAG-tagged Tie2ΔECD was treated with 75 μM $KBrO_3$ in serum-free media for 24 hours and analyzed as in Figure 4A.

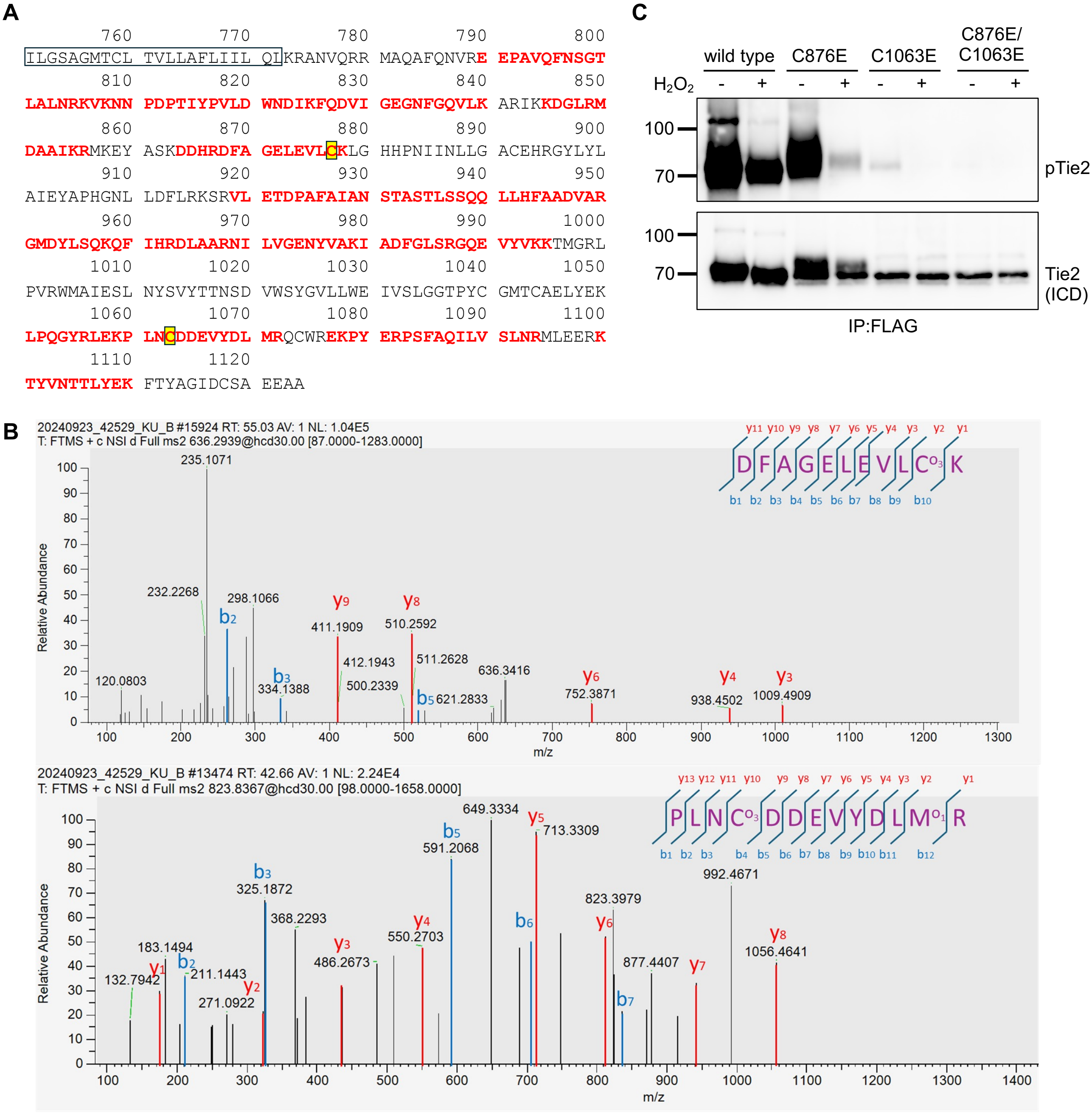

Supplementary Figure S10. (A) Tie2ΔECD peptide sequence with fragment identified by mass spectrometry indicated in red and two modified cysteines, C876 and C1063, highlighted. Box represents amino acid sequences of transmembrane domain. (B) Annotated MS2 chromatogram of the tri-oxidated cysteine containing peptides. Respective peptide sequences are shown right above. Tri- and mono- oxidation are denoted with O3 and O1. (C) FLAG-tagged Tie2ΔECD and its mutants transfected into HEK293T cells were precipitated using anti-FLAG antibody and blotted using anti-pTie2 and anti-Tie2 (ICD) antibodies.

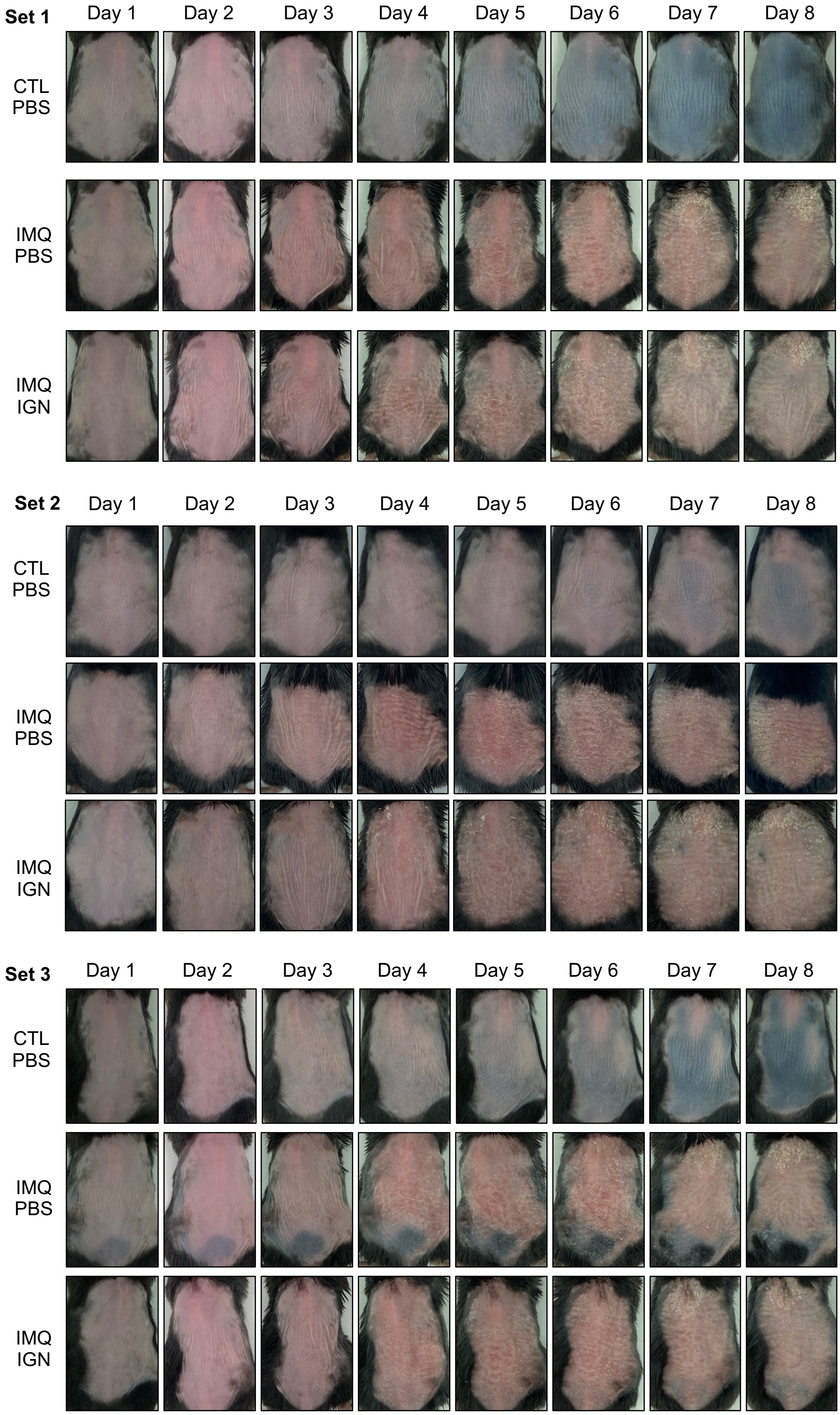

Supplementary Figure S11. Representative back skin images of mice treated with a cream application for seven consecutive days and subcutaneous injection of either PBS or IGN-002 (10 mg/kg) every two days. Images were taken right before cream application.

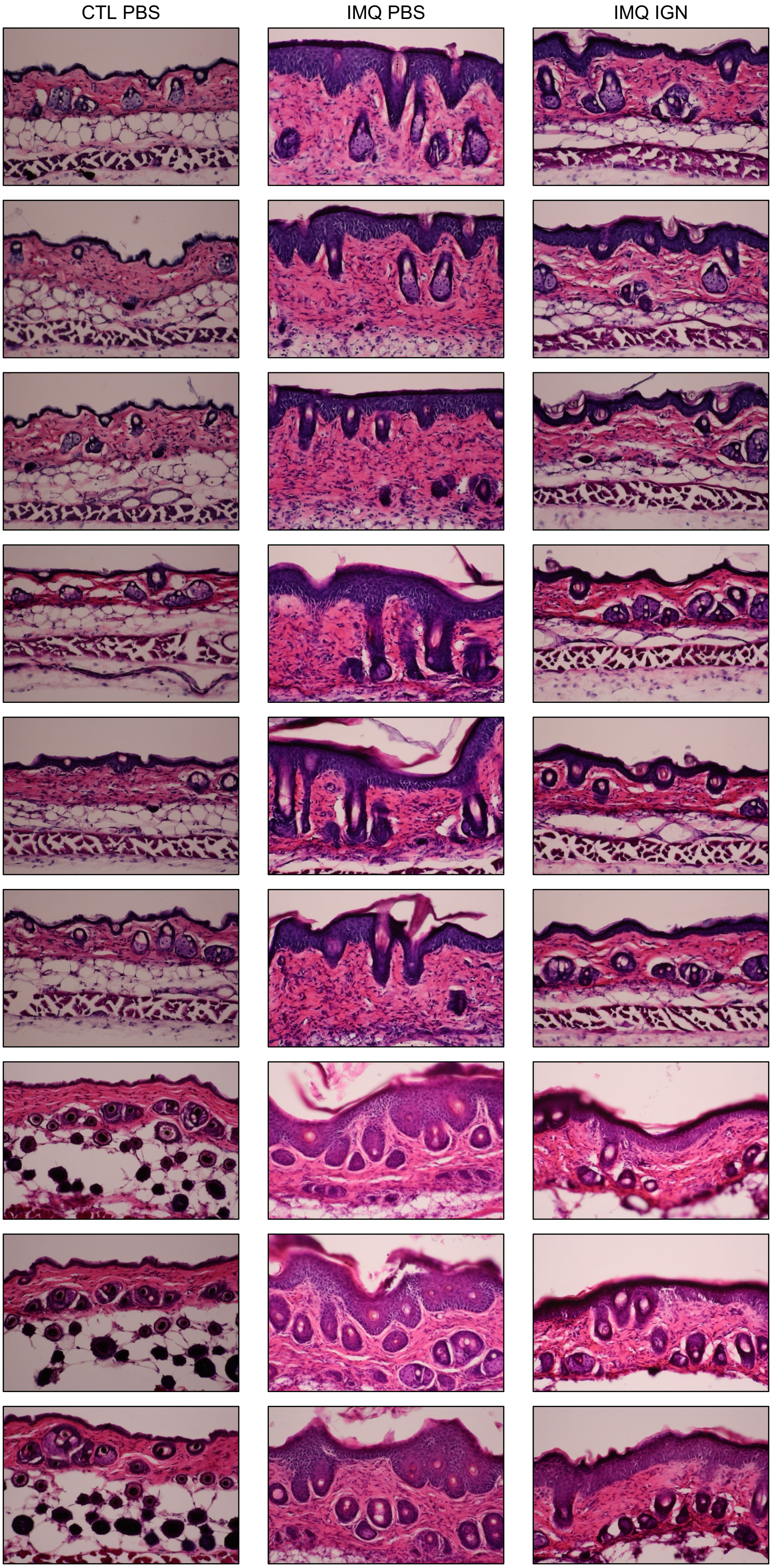

Supplementary Figure S12. Hematoxylin and eosin staining of mouse back skin after seven days of combined treatment. 20× magnification.
